# Between Joy and Sympathy: Smiling and Sad Recipient Faces Increase Prosocial Behavior in the Dictator Game

**DOI:** 10.3390/ijerph18116172

**Published:** 2021-06-07

**Authors:** Martin Weiß, Grit Hein, Johannes Hewig

**Affiliations:** 1 Translational Social Neuroscience Unit, Center of Mental Health, Department of Psychiatry, Psychoso-matic and Psychotherapy, University of Würzburg, 97080 Würzburg, Germany; hein_g@ukw.de; 2 Department of Psychology I: Differential Psychology, Personality Psychology and Psychological Diagnostics, Institute of Psychology, University of Würzburg, 97070 Würzburg, Germany; hewig@psychologie.uni-wuerzburg.de

**Keywords:** emotional influence, dictator game, facial expression, social decision-making

## Abstract

In human interactions, the facial expression of a bargaining partner may contain relevant information that affects prosocial decisions. We were interested in whether facial expressions of the recipient in the dictator game influence dictators’ behavior. To test this, we conducted an online study (*n* = 106) based on a modified version of a dictator game. The dictators allocated money between themselves and another person (recipient), who had no possibility to respond to the dictator. Importantly, before the allocation decision, the dictator was presented with the facial expression of the recipient (angry, disgusted, sad, smiling, or neutral). The results showed that dictators sent more money to recipients with sad or smiling facial expressions and less to recipients with angry or disgusted facial expressions compared with a neutral facial expression. Moreover, based on the sequential analysis of the decision and the interaction partner in the preceding trial, we found that decision-making depends upon previous interactions.

## 1. Introduction

Classical economic theories such as rational choice theory [1] explain economic behavior as a function of maximizing personal utility. Consequently, monetary allocations in experimental paradigms such as the dictator game (*DG*) are assumed to reflect the striving for maximum payoffs. In the *DG*, the proposer is asked to keep an arbitrary share of a sum of money and offer the rest to the recipient, who has no possibility of action. In contrast, the ultimatum game (UG) has a strategic component (see, e.g., [2]) as the recipient can reject the offer, leaving both players without money. Although participants in the *DG* offer less money compared with proposers in the UG, they still deviate from rational choice [3]. Due to the absence of a fear of rejection in the *DG*, offering money to the recipient can be motivated by altruistic or (pro)social motives [4]. Altruism as “a motivational state with the ultimate goal of increasing another’s welfare” [5] is based on empathic concern (EC) for the well-being of others [6]. Altruistic and prosocial behavior might even foster own well-being, happiness [7], and (mental) health [8]. Research on a variety of (neuro)psychological variables challenges traditional views on economic behavior, as simple characteristics such as gender [9] or attractiveness [10,11], but also fairness considerations [12], emotional states ([13,14], robotic emotional expressions (see, e.g., [15]), and even personality traits [16,17,18] can influence economic decision-making. In the present study, we extended previous findings by investigating the interplay between emotional states and trait EC and how they affect altruistic behavior in the *DG*.

A central aspect of social behavior is facial expression, as it contains important information that can influence an interaction [19]. Not only emotional states can be inferred from facial expressions [20], but also information about intentions, personality, and character traits [21]. A smiling facial expression of a social interaction partner leads individuals to infer intentions such as trust, cooperation, or affiliation, which facilitates cooperative behavior [22,23,24]. In the UG, participants (as recipients) showed higher acceptance rates after seeing smiling faces of the proposer compared with neutral faces. In turn, angry proposer faces led to even lower acceptance rates than neutral ones [13]. When used as feedback stimuli of the proposer following acceptance or rejection by the recipients, smiling and sad emotional facial expressions led to a subsequent increase in acceptance rates in the UG [25,26]. In contrast, induced feelings of disgust significantly increased rejection rates of unfair offers compared with sad and neutral moods [27]. These studies focused on mutual interactions in which both partners have options for action (i.e., reacting to fair and unfair offers in the UG). However, in everyday life, we also encounter situations without interdependent relationships in the sense of an exchange of goods or social interactions. In the present study, we applied the dictator game to shed more light on the influence of emotional states on unilateral economic decisions.

Empathy describes an individual’s ability to feel the emotional state of another person’s current situation [28]. Building on two distinct factors of cognitive and affective empathy [29,30,31,32], empathy is a multidimensional psychological concept [33]. Important facets of empathy are personal distress in response to perceiving the other person’s distress, EC (such as sympathy), and perspective taking [34]. These facets can lead to distinct emotional and behavioral outcomes [35,36]. Well-evidenced theories [37] emphasize a strong link between affective empathy and altruism. The positive relationship between affective empathy and altruism has also been demonstrated in recent studies on economic decision-making [38,39,40,41]. Based on previous findings and interpretations on the influence of emotional faces in economic games [25,26], we focused on EC in the present study. The relationship between empathy and different emotional faces is supported by a common brain region in the left dorsal inferior frontal gyrus that is associated with trait empathy across different emotional faces [42]. However, there are specific brain areas reflecting the link between empathy and each distinct emotion. Accordingly, we used happy, sad, angry, and disgusted expressions in order to examine their impact on *DG* offers in interactions with empathy-related traits.

In addition, we aimed to investigate prosocial habits by applying a single-trial analysis that considered previous decisions and previous interaction partners as predictors of behavior in the current trial. The concept of prosocial habits is related to the social heuristics hypothesis, which postulates that other-oriented behavior in anonymous one-shot games originates from intuitive processes. These processes are characterized by successful strategies in social interactions and the internalization of norms [43], such as the norm of reciprocity [44]. Specifically, in one-shot interactions with different strangers, the concept of generalized reciprocity [45] might play a crucial role. Generalized reciprocity applies to situations in which a person who has been treated positively or negatively by others in the past treats another person according to the same principle, commonly referred to as “paying it forward” [46]. Past experiences either refer to experiences outside the experimental context or to one’s behavior in previous trials of the experiment. We expected that emotional facial expressions would activate generalized reciprocity in different ways, depending on the subjective value of the counterpart’s emotion to the decision-maker (and probably also the preceding interaction partner’s emotion).

To the best of our knowledge, there has been no study to date that has closely examined the interplay between trait empathy and affective faces representing the counterpart in a social decision context. Based on the theoretical considerations of altruistic behavior in the *DG*, its relationship with EC, and the influence of emotional faces on decision-making, we derived the following hypotheses: We assumed that smiling and sad recipient faces would lead to more prosocial decisions by dictators compared with a neutral facial expression [13,25,26]. Moreover, we expected a main effect of trait EC on prosocial behavior [41] as well as an interaction between trait EC and sad faces, as these faces might elicit sympathy in the dictator [25]. Building on previous literature on prosocial habits and trial-by-trial analyses [47,48], we examined whether the face of the preceding recipient and the participants’ preceding decision might influence decision-making in the current trial.

## 2. Materials and Methods

### 2.1. Participants

A priori estimation of the required sample size was conducted with G-power software [49]. An assumed average effect of *r* = 0.35 for affective empathy on prosocial behavior in the *DG* [41], α = 0.05, and β = 0.95, yielded a required sample size of *n* = 94. Of 113 participants who started the task (see below), 1 person did not complete the entire task and 6 persons were excluded due to extremely fast completion times, so that the final sample for data analysis included 106 participants (78 women, mean age = 30.48 years, SD = 13.46). Participants either received a course credit or participated without compensation.

### 2.2. Experimental Procedure

First, participants were asked to complete questionnaires on demography and trait empathy. We used a German version [50] of the Interpersonal Reactivity Index (IRI [31]) with 16 items on a 5-point Likert scale. The questionnaire includes four subscales (with four items each) of empathy, which are perspective taking (PT), empathic concern (EC), fantasy (FS), and personal distress (PD). Afterward, participants were informed that the study was part of a larger project and that they would prepare offers for participants who had played a similar game previously and provided photos of themselves. After selecting a one-shot dictator offer for a recipient without facial expression (i.e., a generic *DG*), participants played 20 rounds of the *DG* with faces. Pictures of the recipients were taken from a validated set of facial expression stimuli [51]. Each trial started with either a smiling, neutral, angry, sad, or disgusted facial expression of the recipient, which we refer to as the identity of the recipient. For each participant, we selected six male and female persons, from which we randomly drew five men and five women for the task and one each for the manipulation check. From each person, one facial expression was randomly selected and assigned to one of the five different identities. Next, participants were asked to select the amount of money they would give to the recipient on a slider measure ranging from EUR 0 to 10. We presented each identity twice to assess the reliability of decision-making toward different identities (Table 1). In the manipulation check, faces were rated on a 7-point Likert scale asking for valence (1 = negative to 7 = positive) and arousal (1 = not exciting at all to 7 = very exciting).

### 2.3. Statistical Analyses

For the analysis of the manipulation check, we conducted two one-way repeated measures analyses of variance with the factor emotion as predictor and the ratings for valence and arousal as criterion, respectively.

To examine the additional value of facial expressions to the generic *DG*, we computed an emotional index (*EI*) for each emotion. The *EI* quantifies enhancing or diminishing effects of the different identities on prosocial behavior (offers).

(1) $$EI_{emotion/\;generic\;DG}=\frac{Offer_{emotion}-Offer_{generic\;DG}}{Offer_{generic\;DG}}$$

A negative EI value indicated that offers in the generic *DG* were more prosocial than toward the respective identity.

For the main analysis, we used two different models with decision as the dependent variable. First, we addressed the main hypotheses on EC and the recipient by computing a general linear model with the predictor identity (levels: smiling, neutral, angry, sad, and disgusted), and standardized EC. For analyses on sequence effects on a single-trial basis, we used a linear mixed-effects model. As level 1 predictors, we included the factor’s identity and the identity of the preceding trial (previous identity). EC and the participant’s decision in the preceding trial (previous decision) were entered as standardized level 2 predictors. We included a random slope for identity and a random intercept for participant. The final model was selected based on the Akaike criterion and the simpler model structure. In this model, no interaction terms including both previous identity and previous decision at the same time were included.

## 3. Results

### 3.1. Manipulation Check

Valence ratings on the faces showed a significant main effect for emotion (*F* = 443.8, *df* = 4, *p* < 0.001) with significant post hoc comparisons for smiling > sad > neutral > angry = disgusted facial expressions. We also found an arousal effect for emotional faces (*F* = 16.5, *df* = 4, *p* < 0.001) with higher ratings for smiling, angry, and disgusted faces compared with neutral (*p*s < 0.001) and smiling compared with sad (*p* = 0.003).

### 3.2. Behavior

Averaged *EIs* indicated that faces did not enhance prosocial behavior per se as all values of *EI* were negative (*EI*_smiling_ = −0.005, *EI*_neutral_ = −0.126, *EI*_angry_ = −0.274, *EI*_sad_ = −0.059, and *EI*_disgusted_ = −0.364).

**Figure 1:**
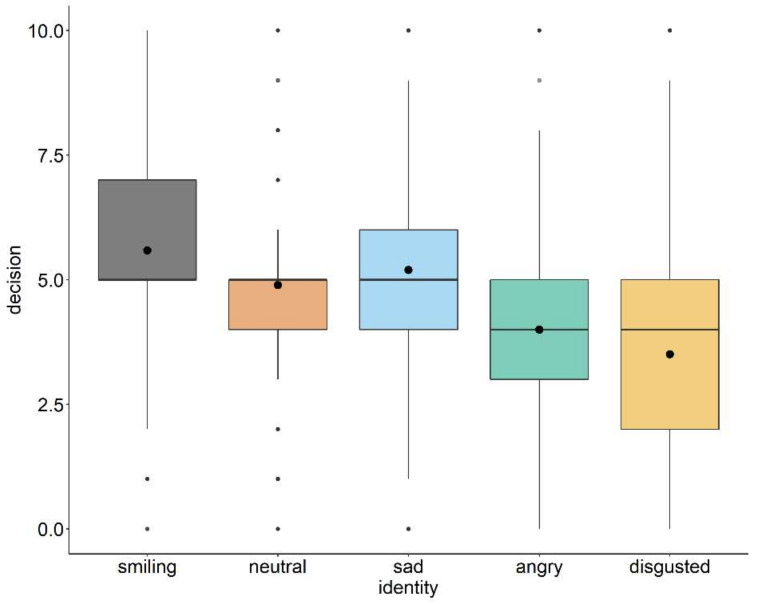
Boxplots of decisions of dictators toward the five different recipient identities. The dots within the boxes indicate the mean per recipient identity.

The general linear model revealed a main effect of identity (Figure 1) with more prosocial behavior toward the smiling (*β* = 0.69, *p* < 0.001) and sad identities (*β* = 0.30, *p* = 0.032), and less prosocial decisions for the angry (*β* = −0.89, *p* < 0.001) and disgusted identities (*β* = −1.39, *p* < 0.001) compared with the neutral identities. In addition, there was a trend for an effect of EC on prosocial behavior (*β* = 0.18, *p* = 0.065), indicating more prosocial decisions with increasingly empathic concern.

**Figure 2:**
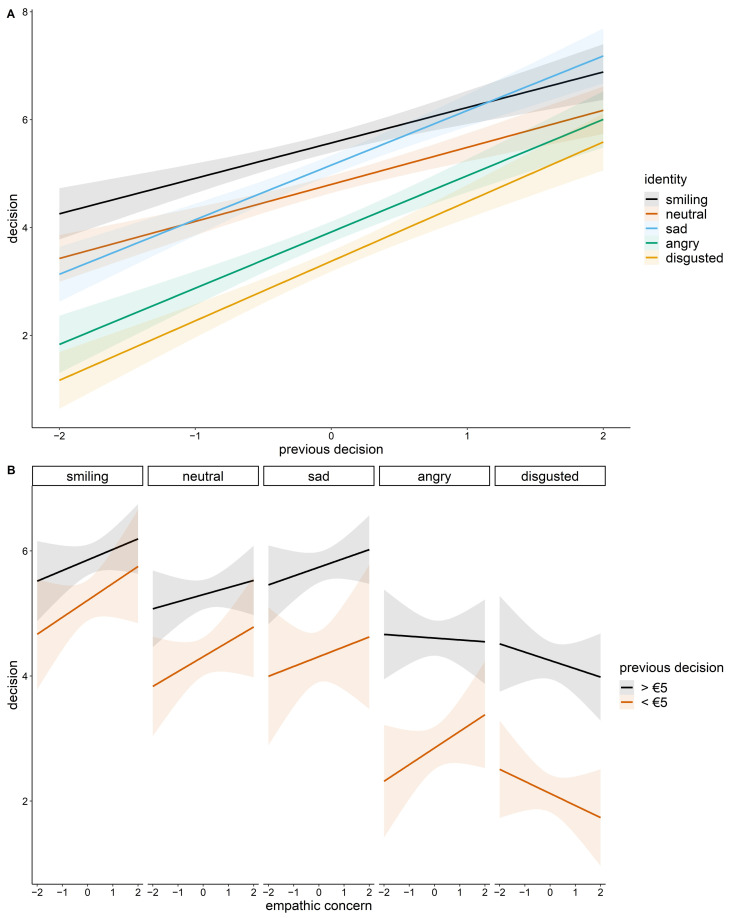
Interactions between fixed effects predictors. (**A**) Two-way interaction between the decision in the preceding trial (previous decision) and recipient identity; (**B**) Three-way interaction between empathic concern, previous decision, and recipient identity. For the illustration of the latter, we used a median split (median = EUR 5) for previous decision. Shaded areas represent the 95% confidence interval.

The single-trial mixed model reflected the main effect of the recipient identity as in the previous model (Figure 1, Table 2). With regard to sequential effects, i.e., the influence of the previous identity and the previous decision on the current decision, we found a main effect of the previous decision, indicating increasingly prosocial behavior in the current trial after prosocial decisions in the preceding trial (*β* = 0.12, *p* = 0.003). The interaction between identity and previous decision revealed that the sad identity received increasingly prosocial offers when participants offered more in the previous trial (*β* = 0.22, *p* = 0.04; Figure 2a, Table 2). The three-way interaction between identity, EC, and previous decision showed that the angry identity especially received decreasingly prosocial offers by empathic participants who showed more prosocial behavior in the preceding trial (*β =* −0.22, *p* = 0.041; Figure 2b, Table 2), whereas the opposite was true if they had given a low offer in the preceding trial. Moreover, we found unspecific interactions of fixed effects, indicating that angry-looking recipients benefited from highly empathic concerning dictators who played with the smiling (*β* = 0.61, *p* = 0.023) or the sad recipient (*β* = 0.54, *p* = 0.040) in the preceding trial. We refrain from further interpretation as the three-way interaction between identity, EC, and the previous identity was not significant. The complete model results can be found in Tables S1 and S2 in the Supplementary Material.

## 4. Discussion

We investigated the interplay between a person’s EC and the recipient’s emotional state as well as its influence on altruistic behavior in a modified dictator game. In line with similar research on emotional states and economic decision-making [13], smiling faces led to increasingly prosocial decisions by the dictators. Giving more to individuals who show a smiling face reflects the internalized norm of generalized reciprocity. Even in the absence of direct reciprocal interactions, individuals are likely to know from their own experience that smiling is a signal of cooperative intentions, even in one-shot interactions [22]. This, in turn, might have caused the rewarding of smiling individuals.

As expected, sad facial expressions also provoked fairer monetary allocations. This result extends previous literature that showed higher acceptance rates for proposers providing sad faces as feedback stimuli after rejected offers in the UG [25,26]. Consequently, sad faces might elicit sympathy and thereby trigger prosocial behavior in economic decision-making. This is consistent with results from marketing research, which demonstrated that individuals donated more to a charity when advertisements showed a child with a sad face as compared to a neutral-faced or happy-faced child [52]. However, these results also indicate that the impact of sympathy depends on the context, since in our experiment, the effect of smiling faces was stronger than that of sad faces. This suggests a difference in the intensity of sympathy elicited in economic games and advertisements targeting charity giving.

In contrast, the negative emotions, anger and disgust, led to avoidance behavior and thus, less prosocial offers [13,27]. Since research showed that negative emotional facial expressions predict non-cooperative decisions [22], participants might also have considered such defective emotional states in our one-shot dictator game, resulting in less prosocial behavior. In our study, the behavioral effects are consistent with the manipulation check results in terms of stimulus valence and not in terms of arousal. This pattern strengthens the notion that valence, rather than arousal alone, contributed to the results. For instance, the behavioral effects toward smiling and angry recipients occurred in opposite directions, just like their valence ratings but contrary to their arousal ratings (see also [13]). However, the beneficial effect of a sad facial expression contradicts other findings showing an overall detrimental effect of negative emotions on decision-making, regardless of the specific nature of the emotion [53].

By presenting the recipient identities twice, we showed that the influence of emotional states was highly reliable. A strength of our paradigm was the possibility to ascribe concrete values to emotions. Our data suggest that in this paradigm, the smiling face was as good as a non-emotional state in the generic *DG*, but adds no value to the generic *DG*. Moreover, neutral and negative emotions even worsen altruistic decisions compared with context-free decisions. This is an issue that should be considered and explored in more detail in future studies.

Although we found a trend for more overall prosocial behavior among high-EC individuals, none of the expected interactions with recipient identities emerged. A possible explanation for the low correlations between EC and altruistic behavior might be the measure of trait empathy. Addressing inconsistent and weak effects of trait empathy [54,55], Edele et al. (2013) used a photo-based measure of empathy with naturalistic stimuli [41]. The authors assumed that their empathy measure is less sensitive to the hindsight bias and thereby can reveal stronger correlations between empathy and altruistic behavior. Moreover, interaction effects between EC and emotional states might be small and would require a larger sample size in order to be detected.

Finally, we investigated sequence effects to elucidate habits of prosocial decision-making. As increasingly prosocial decisions in the preceding trial led to more prosocial decisions for the sad face in the current trial, economic decisions with different proposer identities seem to depend on each other. Possibly, individuals in an altruistic mode (i.e., with prosocial decisions in the previous trial) are triggered even more by the sad facial expression. By contrast, angry faces interrupt this mode, especially when individuals have a higher EC. The unspecific fixed effects interactions with the preceding identity indicate that smiling and sad faces of the previous interaction facilitate altruistic behavior toward angry recipients in the current trial, especially when the dictator has a higher level of EC. Pointing to another beneficial value of smiling and sad facial expressions for prosocial decisions, these effects need further investigation and possibly a larger sample size. In addition, as we had no explicit hypothesis and only 20 trials with five different emotional states, we did not analyze the influence of preceding decisions and identities that occurred more than one round earlier, which may be a valuable adaption in future studies. As suggested in recent research [47], economic decisions are not necessarily independent of each other, a finding that has so far been of secondary interest.

We briefly address the limitations of the current study and ideas for potential further developments within this line of research. The reliability of EC was considerably lower, for example, compared with PT. However, other studies also reported lower reliabilities for either affective or cognitive empathy (e.g., Cronbach’s alpha = 0.66 for cognitive empathy compared with Cronbach’s alpha = 0.75 for affective empathy in [41]). The effect size assumed a priori was estimated rather optimistically (medium-sized effect). A replication of the study with a more conservative estimate based on a smaller effect may reveal more subtle mechanisms that were not detectable here. We only included the relevant negative emotions for economic decision-making behavior based on previous findings in the present study. Although the validation paper of the Radboud Face Database [51] reports an 82% agreement rate for intended and attributed emotional expressions, the authors also noted that, inter alia, intended disgust was sometimes mistaken for either anger or contempt. Therefore, the examination of other universal emotions (e.g., fear, contempt, and surprise) in an economic context would require a more complex design with a comprehensive stimulus evaluation to identify potential overlapping effects. The generalizability is limited due to the high proportion of female participants. Future studies can specifically aim at sex effects with equally distributed participants. As participants were not incentivized with real money in an economic task, further research is needed to compare the influence of real versus hypothesized rewards on the presented effects. Future studies might counterbalance the order of the empathy measurement and the dictator game. Completing the trait empathy questionnaire prior to the *DG* might have accentuated empathy-related behaviors for participants. To rule out order effects within the *DG*, one or more trials of the generic *DG* can be placed in-between emotional trials.

## 5. Conclusions

Our results show that emotional expressions of an interaction partner can influence economic decision-making, even when direct reciprocity is not possible. Thus, people try to adapt by acting in habitual patterns to maximize the chance of acting appropriately in a given situation. Smiling and sad faces make people behave more altruistically, whereas angry and disgusted faces achieve the opposite. The present findings indicate small effects of trait EC on the interplay between emotional states and prosocial behavior. Extensions and adaptions of the current paradigm and possibly alternative trait measures are needed in future experiments.

## Figures and Tables

**Table 1:** Correlations and descriptive statistics (M and SD) for the subscales of trait empathy and the dictator offers for the different recipients and the generic dictator game. Reliabilities (ω) are displayed on the diagonal, except for the one-shot generic dictator game.

	1	2	3	4	5	6	7	8	9	10
FS (1)	0.74									
EC (2)	0.42	0.63								
PD (3)	0.06	0.25	0.82							
PT (4)	0.20	0.22	−0.19	0.80						
smiling (5)	0.01	0.12	0	−0.02	0.96					
neutral (6)	0.07	0.12	−0.15	0.05	0.61	0.96				
sad (7)	−0.04	0.13	0.04	0.06	0.63	0.61	0.95			
angry (8)	−0.06	0.06	−0.07	0.07	0.25	0.69	0.57	0.95		
disgusted (9)	−0.07	−0.04	−0.13	0.08	0.28	0.62	0.52	0.81	0.97	
generic (10)	−0.04	0.01	0	−0.14	0.44	0.43	0.31	0.22	0.17	
M	3.48	3.70	2.70	3.60	5.59	4.89	5.20	4.00	3.50	5.98
SD	0.70	0.55	0.73	0.67	1.71	1.59	1.83	1.93	2.06	2.01

Note: FS = fantasy scale; EC = empathic concern; PD = personal distress; PT = perspective taking.

**Table 2:** Intercept and significant fixed effects for the trial-by-trial analysis on dictator behavior.

Fixed Effects	Values	SE	*t*-Value	*p*-Value
(intercept)	4.64	0.14	34.11	<0.001
smiling	0.73	0.15	4.94	<0.001
sad	0.29	0.15	1.99	0.049
angry	−0.86	0.14	−6.13	<0.001
disgusted	−1.39	0.15	−9.06	<0.001
previous decision	0.12	0.04	2.93	0.003
sad × previous decision	0.22	0.11	1.97	0.049
angry × previous decision × EC	−0.22	0.11	−2.05	0.041

Note: Baseline category = neutral identity.

## Data Availability

The data that support the findings of this study are openly available at https://osf.io/dsuqa (accessed on 7 May 2021).

## Supplementary Materials

The following are available online at https://www.mdpi.com/article/10.3390/ijerph18116172/s1, Table S1: Fixed-effects omnibus test for the trial-by-trial analysis on dictator behavior, Table S2: Intercept and fixed-effects parameters for the trial-by-trial analysis on dictator behavior.
