# Supplementary material for: Between Joy and Sympathy: Smiling and Sad Recipient Faces Increase Prosocial Behavior in the Dictator Game
Source: Int J Environ Res Public Health. 2021 Jun 7;18(11):6172. doi: 10.3390/ijerph18116172 (PMC8201160; doi:10.3390/ijerph18116172)
Supplement: Supplementary file 1 [file ijerph-18-06172-s001.zip › ijerph-1232729-supplementary.pdf]

Supplementary Materials

Table S1. Fixed effects omnibus test for the trial-by-trial analysis on dictator behavior

	F-value	Num DF	*p*-value
Identity	26.15	4	< .001
EC	.75	1	.388
previous identity	.74	4	.565
previous decision	8.59	1	.003
identity × previous identity	.65	16	.846
identity × EC	.92	4	.453
previous identity ×EC	.45	4	.773
identity × previous decision	4.12	4	.003
EC × previous decision	.08	1	.773
identity × previous identity × EC	.80	16	.688
identity × previous decision × EC	5.35	4	< .001

Note. EC = empathic concern; Num DF = number of degrees of freedom

Table S2. Intercept and fixed effects parameters for the trial-by-trial analysis on dictator behavior

Fixed effects	Values	SE	*t*-value	*p*-value
(intercept)	4.64	.14	34.11	< .001
smiling	.73	.15	4.94	< .001
sad	.29	.15	1.99	.049
angry	-.86	.14	-6.13	< .001
disgusted	-1.39	.15	-9.06	< .001
previous_smiling	.01	.08	.10	.921
previous_sad	.05	.08	.57	.569
previous_angry	.11	.08	1.30	.193
previous_disgusted	.12	.09	1.39	.166
EC	.12	.14	.87	.388
previous decision	.12	.04	2.93	.003
smiling × previous_smiling	.11	.27	.41	.678
smiling × previous_sad	-.33	.26	-1.25	.212
smiling × previous_angry	-.02	.25	-.10	.922
smiling × previous_disgusted	-.13	.27	-.49	.626
sad × previous_smiling	.18	.26	.69	.492
sad × previous_sad	-.27	.27	-1.01	.312
sad × previous_angry	.03	.26	.11	.915
sad × previous_disgusted	.33	.28	1.16	.245
angry × previous_smiling	.12	.26	.46	.648
angry × previous_sad	-.22	.26	-.82	.412
angry × previous_angry	.00	.27	-.01	.989
angry× previous_disgusted	-.13	.27	-.47	.637
disgusted × previous_smiling	-.10	.26	-.37	.711
disgusted × previous_sad	-.31	.26	-1.19	.235
disgusted × previous_angry	-.16	.27	-.60	.549
disgusted × previous_disgusted	-.01	.28	-.04	.968
EC × previous decision	.01	.04	.29	.773
smiling × EC	.03	.15	.21	.835
sad × EC	.05	.15	.32	.752

angry × EC	-.02	.14	-.15	.881
disgusted × EC	-.23	.16	-1.50	.137
previous_smiling × EC	.00	.09	.00	.999
previous_sad × EC	.04	.08	.43	.665
previous_angry × EC	.10	.09	1.10	.270
previous_disgusted × EC	.08	.09	.87	.382
smiling × previous decision	.18	.11	1.67	.096
sad × previous decision	.22	.11	1.97	.049
angry × previous decision	-.18	.10	-1.80	.072
disgusted × previous decision	.10	.11	.97	.332
smiling × previous_smiling × EC	.46	.30	1.54	.123
smiling × previous_sad × EC	.26	.26	.99	.324
smiling × previous_angry × EC	.17	.26	.66	.512
smiling × previous_disgusted × EC	-.02	.28	-.07	.945
sad × previous_smiling × EC	.35	.26	1.31	.190
sad × previous_sad × EC	.03	.28	.12	.908
sad × previous_angry × EC	.09	.26	.36	.721
sad × previous_disgusted × EC	.25	.29	.86	.387
angry × previous_smiling × EC	.61	.27	2.28	.023
angry × previous_sad × EC	.54	.26	2.06	.040
angry × previous_angry × EC	.52	.29	1.81	.070
angry× previous_disgusted × EC	.34	.27	1.26	.209
disgusted × previous_smiling × EC	.52	.28	1.87	.062
disgusted × previous_sad × EC	.28	.28	1.01	.314
disgusted × previous_angry × EC	.39	.29	1.34	.179
disgusted × previous_disgusted × EC	.37	.30	1.23	.218
smiling × previous decision × EC	-.10	.11	-.92	.358
sad × previous decision × EC	.19	.12	1.60	.109
angry × previous decision × EC	-.22	.11	-2.05	.041
disgusted × previous decision × EC	.14	.11	1.23	.221

Note. EC = empathic concern
